# Reference values of mitral and tricuspid annular plane systolic excursion for the evaluation of left and right ventricular performance

**DOI:** 10.1186/1532-429X-14-S1-M3

**Published:** 2012-02-01

**Authors:** Florian Andre, Dirk Lossnitzer, Sebastian Buss, Henning Steen

**Affiliations:** 1 Department of Cardiology, University of Heidelberg, Heidelberg, Germany

## Background

Mitral annular plane systolic excursion (MAPSE) and tricuspid annular plane systolic excursion (TAPSE) correlate with the left and right ventricular (LV,RV) ejection fraction as well as with the prognosis of patients with heart failure. Furthermore, M/TAPSE are sensitive markers for impaired longitudinal function which can be the earliest marker of myocardial dysfunction in cardiomyopathies with preserved ejection fraction. Cardiac MRI is the reference method for ventricular function and morphology and both M/TAPSE can be easily obtained from cine images. To date, there are no age and gender specific reference values for M/TAPSE derived from a healthy collective of normal volunteers.

In this study we want to provide reference values for M/TAPSE for cardiac MRI with regard to age and gender.

## Methods

60 male and 59 female healthy volunteers were divided into three age groups (I=20-34yrs, II=35-49yrs, III≥50yrs). Images were acquired on a 1.5T whole body MRI scanner (Philips Achieva) using a SSFP sequence of short axis slices covering the entire left ventricle as well as two-, three- and four-chamber views. M/TAPSE were measured on 4-chamber SSFP images. Two separate reference lines were drawn in diastole and systole from the basal lateral tricuspid (TAPSE) and the basal anterior mitral leaflet (MAPSE) to a reference point on the left chest surface. The lengths differences of the reference lines between diastole and systole were measured and represent both M/TAPSE. P<0.05 was considered significant.

## Results

The normal values for MAPSE and TAPSE are shown in table 1 and 2. Collectively, there were no significant differences between the different age and gender groups. Only in the subgroup analyses of the MAPSE values, the difference between men of group I and II and the discrepancy between men and women in group II were significant (p<0.05).

**Table 1:** MAPSE

	Male	Female	Both genders
Group I	15.6±6.2	14.9±5.8	15.2±5.9
Group II	12.8±7.3	15.7±8.1	14.3±8.2
Group III	14.8±7.9	13.1±7.0	14.0±7.5
All groups	14.4±7.4	14.6±7.2	

Values are given in mm ± 2 standard deviations.

**Table 2:** TAPSE

	Male	Female	Both genders
Group I	22.1±12.7	20.9±8.4	21.5±10.7
Group II	19.3±7.3	21.5±9.4	20.4±8.6
Group III	20.6±12.3	18.4±6.9	19.6±10.1
All groups	20.7±11.1	20.3±8.6	

Values are given in mm ± 2 standard deviations.

## Conclusions

In this study, we present age and gender specific reference values of longitudinal LV and RV function in a large group of normal volunteers. Interestingly, in healthy volunteers the values of M/TAPSE are only marginally influenced by age or gender. However, further investigations are needed to confirm these findings and to provide quantification of pathological values.

## Funding

None.

